# Antitumor Activity of Ethanolic Extract of *Dendrobium formosum* in T-Cell Lymphoma: An *In Vitro* and *In Vivo* Study

**DOI:** 10.1155/2014/753451

**Published:** 2014-05-18

**Authors:** Ritika Prasad, Biplob Koch

**Affiliations:** Department of Zoology, Banaras Hindu University, Varanasi 221005, India

## Abstract

*Dendrobium*, a genus of orchid, was found to possess useful therapeutic activities like anticancer, hypoglycaemic, antimicrobial, immunomodulatory, hepatoprotective, antioxidant, and neuroprotective activities. The study was aimed to evaluate the anticancer property of the ethanolic extract of *Dendrobium formosum* on Dalton's lymphoma. *In vitro* cytotoxicity was determined by MTT assay, apoptosis was determined by fluorescence microscopy, and cell cycle progression was analysed using flow cytometry; *in vivo* antitumor activity was performed in Dalton's lymphoma bearing mice. The IC_50_ value of ethanolic extract was obtained at 350 **μ**g/mL in Dalton's lymphoma cells. Fluorescence microscopy analysis showed significant increase in apoptotic cell death in dose- and time-dependent manner which was further confirmed through the resulting DNA fragmentation. Further, flow cytometry analysis showed that the ethanolic extract arrests the cells in G_2_/M phase of the cell cycle. The *in vivo* anticancer activity study illustrates significant increase in the survival time of Dalton's lymphoma bearing mice on treatment with ethanolic extract when compared to control. These results substantiate the antitumor properties of ethanolic extract of *Dendrobium formosum* and suggest an alternative in treatment of cancer. Further studies are required regarding the isolation and characterization of bioactive components along with the analysis of molecular mechanism involved.

## 1. Introduction

Nature is the most valuable source of therapeutic compounds as enormous chemical diversity is present in millions of species of plants, animals, marine organisms, and microorganisms [1]. Today, cancer is one of the leading causes of death worldwide. In a report by Siegel et al. in 2013, a total of 1,660,290 new cancer cases and 580,350 deaths from cancer were predicted in the United States in 2013 [2]. Cancer chemoprevention was first defined as “a strategy of cancer control by administration of synthetic or natural compounds to reverse or suppress the process of carcinogenesis” [3]. Nowadays drugs obtained from medicinal plants play a crucial role in the treatment of cancer and most of the plant secondary metabolites and their derivatives have been applied to combat cancer [4, 5].

Orchids are ornamental plants and they are also well known for their medicinal value. They belong to the family Orchidaceae, with approximately 20,000 species and more than 850 genera. A total of 365 plants, including several orchids, are listed in the earliest known Chinese Materia Medica [6]. In India, the northeastern states are renowned hot spot of orchids with approximately 876 orchid species in 151 genera, which constitutes about 70% of total orchids in India. The local tribe of this region makes use of several orchid plants for variety of folk medicines to cure as they are found to be rich in flavonoids, glycosides, carbohydrates, and other phytochemical contents [7]. Extracts prepared and metabolites isolated from the orchid plants were found to possess useful therapeutic activities like diuretic, antirheumatic, anti-inflammatory, anticarcinogenic, hypoglycaemic, antimicrobial, and neuroprotective activities [8].* Dendrobium* is the largest genus of orchids, containing 1,200 species. The* Dendrobium* genus also possesses immunomodulatory, hepatoprotective, antioxidant, anticancer, and neuroprotective activities. Medicinal plants from* Dendrobium* genus are highly valued, and therefore methodologies are being developed to validate* Dendrobium* derived drugs for their therapeutic use [9]. In a study by Ho and Chen, it has been reported that* Dendrobium *species possess anticancer activity. Their group has found that moscatilin, extracted from the stems of* Dendrobium loddigesii*, shows potent cytotoxicity against cancer cell lines derived from different tissue origins [10]. Erianin, a natural product extracted from* Dendrobium chrysotoxum,* inhibits the growth of HL-60 cells [11]. The ethanolic extract of stems of* Dendrobium nobile *was found to exhibit significant antioxidant activity [12]. The antitumor and antibacterial activities of* Dendrobium nobile *extract have also been reported [13]. After acquiring knowledge about the anticancer activity of different* Dendrobium species*, we identified and selected the plant,* Dendrobium formosum, *as there are no reports of its antitumor activity till now. To the best of our knowledge, this is the first study to demonstrate the antitumor activity of* Dendrobium formosum*.

## 2. Materials and Methods

Roswell Park Memorial Institute 1640 (RPMI-1640) medium, fetal bovine serum (FBS) antibiotic solution (penicillin 1000 IU and streptomycin 10 mg/mL), and MTT (3-(4,5-dimethylthiazol-2-yl)-2, 5 diphenyltetrazolium bromide dye) were purchased from [Himedia, India], whereas DMSO (dimethyl sulphoxide), RNAse, and proteinase K were obtained from GeNie, Merck, India. Ethidium bromide, Triton X-100, and other chemicals of analytical grades were purchased from Lobachemie Pvt. Ltd., India. Acridine orange was obtained from SRL Pvt. Ltd., India, SDS (sodium dodecyl sulphate) was obtained from Bio basic Inc., India, and agarose was purchased from Sigma, USA, whereas propidium iodide (PI) was obtained from EMD Millipore-Calbiochem, USA.

### 2.1. Collection of Plant Material

From the genus* Dendrobium, Dendrobium formosum *was identified and collected from Northeastern state of India (Meghalaya) which is rich in plant diversity.

### 2.2. Preparation of the Ethanolic Extract

Leaves of* Dendrobium formosum* (*D. formosum*) were cleaned, air-dried, and grinded. The dried powder obtained was suspended in absolute ethanol (250 mL) at room temperature for 6-7 days. After this, it was filtered through a filter paper and the filtrate was allowed to evaporate to reduce it into the form of residue. This extract (residue) was collected and stored at 4°C and dissolved in fresh distilled water immediately before use [14].

### 2.3. Cell Culture

A murine transplantable T-cell lymphoma of spontaneous origin, designated as Dalton's lymphoma, was used as a tumour model. This tumour was initially originated in the thymus gland of a DBA/2 mouse at the National Cancer Institute, Bethesda, USA, in 1947 and afterwards serially transplanted in the intraperitoneal cavity from mouse to mouse [15].

Dalton's lymphoma (DL) cells harvested from Dalton's lymphoma bearing mice were cultured in RPMI-1640 medium supplemented with 10% FBS and antibiotic solution (penicillin 1000 IU and streptomycin 10 mg/mL) in 5% CO_2_ incubator at 37°C.

### 2.4. Animal Model

For* in vivo* studies Swiss albino mice were taken, which were housed in well-ventilated cages and fed with standard mouse feed and water ad libitum. The animals were acclimatized to standard environmental conditions of temperature (22°C ± 5°C) for 12 h light-dark cycles throughout the experimental period. The animals used for the study were approved by the central animal ethical committee (CAEC) of the university and the ethic number (Dean/12-13/CAEC/210).

*Tumour Model.* The anticancer effect of the ethanolic extract was determined on Dalton's lymphoma, for which ascites tumour was maintained in mice. 1 × 10^6^ cells/mL were transplanted in the peritoneal cavity of the mice. Dalton's lymphoma ascites (DLA) cells can be propagated as transplantable ascites tumour in Swiss albino mice.

### 2.5. Isolation of Mouse Bone Marrow Cells

Bone marrow cells were isolated from femur bones of approximately 8–10-week-old mice by cervical dislocation after mild anaesthesia exposure. The bone marrow was flushed with prewarmed phosphate buffer saline (PBS) through a 24-gauge needle and single cell suspension was prepared by agitation. The cell suspension was centrifuged at 1500 rpm for 5 minutes. The cells were finally resuspended in RPMI-1640 medium supplemented with 10% FBS and antibiotic solution. Cells were seeded in culture plates with supplemented RPMI-1640 medium and maintained in 5% CO_2_ at 37°C for 24 h for the experiment.

### 2.6. MTT Assay

Evaluation of cytotoxicity was done using MTT (3-(4,5-dimethylthiazol-2-yl)-2, 5 diphenyltetrazolium bromide dye) assay in Dalton's lymphoma (DL) and normal mouse bone marrow cells. DL cells were harvested from DL bearing mice and the mouse bone marrow cells were isolated from the femur bone of a normal adult mouse. 2.5 × 10^4^ cells/mL DL cells and bone marrow cells (BMC) were seeded in RPMI 1640 medium (10% FBS and antibiotic solution) in a culture plate with different concentrations (200 *μ*g/mL to 600 *μ*g/mL) of* D. formosum* ethanolic extract along with a vehicle and a control sample. The culture plates were incubated for 24 h at 37°C and 5% CO_2_. After incubation, 10 *μ*L of MTT (5 mg/mL in PBS) was added to each well and it was incubated for additional two hours at 37°C to allow intracellular reduction of the soluble yellow MTT to insoluble purple formazan crystals. These formazan crystals formed were dissolved in 100 *μ*L of DMSO and incubated for 30 minutes at 37°C [16]. The absorbance of the solution was read at 570 nm using a microplate reader (Microscan (MS5608A), India). Three independent experiments were carried out and five replicates were taken for each experiment. Concentration of ethanolic extract of* Dendrobium formosum* resulting in 50% reduction of cell viability, inhibitory concentration (IC_50_ value), was considered by the formula mentioned below:

(1) $$\begin{array}{c} \%\;\text{inhibition}=\frac{\text{Control}\;\text{abs}-\text{sample}\;\text{abs}}{\text{Control}\;\text{abs}}\times 100. \end{array}$$

### 2.7. Cell Morphology Analysis by Fluorescent Staining

The apoptotic features like chromatin condensation, nuclear shrinkage, and formation of apoptotic bodies can be seen under fluorescence microscope after staining of nuclei with DNA-specific fluorochrome like propidium iodide (PI) [17].

Briefly, DL cells were isolated from DL bearing mouse and washed with PBS. 1 × 10^6^ cells/mL were treated with 50 *μ*g/mL, 100 *μ*g/mL, 150 *μ*g/mL, 200 *μ*g/mL, and 250 *μ*g/mL of the* D. formosum* ethanolic extract for 3 h, 6 h, 16 h, and 20 h at 37°C and 5% CO_2_. The cells were then fixed with absolute ethanol at −20°C for 15 minutes. After fixation, cells were washed and stained with 1 mg/mL propidium iodide (PI) at 37°C for 15 minutes. The cells were washed again and 10 *μ*L cell suspension was taken on a slide. Fluorescent images were scanned using fluorescence microscope (Nikkon E800, Japan) and the images were captured by a digital camera. Similarly, to investigate apoptosis or necrosis, acridine orange (AO) and ethidium bromide (EB) staining method was performed. Acridine orange permeates all the cells and makes the nuclei appear green. Ethidium bromide is only taken up by dead cells when cytoplasmic membrane integrity is lost and the nucleus stains yellowish orange. Therefore, live cells have a normal green nucleus; early apoptotic cells show bright green/yellowish nucleus with condensed or fragmented chromatin; late apoptotic cells display condensed and fragmented orange/red chromatin while the cells that have died from direct necrosis have a structurally normal deep orange nucleus [18].

DL cells were isolated as mentioned above and treated with 50 *μ*g/mL, 100 *μ*g/mL, 150 *μ*g/mL, 200 *μ*g/mL, and 250 *μ*g/mL of the ethanolic extract. The treated cells were incubated for 3 h, 6 h, 16 h, and 20 h at 37°C and 5% CO_2_ after the incubation cells were washed in PBS. Subsequently, the cells were stained with 20 *μ*L ethidium bromide (100 *μ*g/mL) and 20 *μ*L acridine orange (100 *μ*g/mL) in a ratio of 1 : 1. After washing, the cells were resuspended in PBS. The cells were then examined on a slide under a fluorescence microscope (Nikkon E800, Japan) and the images were captured [19]. Quantitative analysis of apoptotic cell death was determined by calculating the apoptotic index (AI). Apoptotic index calculated the frequency of apoptotic cells in dose- and time-dependent manner with respect to control. At least 400 cells were scored under fluorescence microscope for each experiment. 

*Apoptotic Index*. Percentage (%) of Apoptotic cells = (total number of apoptotic cells/total number of cells counted) × 100.

### 2.8. DNA Fragmentation Assay

For* in vitro* DNA fragmentation assay, DL cells (1 × 10^6^ cells/mL) were incubated with 50 *μ*g/mL, 100 *μ*g/mL, 150 *μ*g/mL, 200 *μ*g/mL, and 250 *μ*g/mL of the ethanolic extract for 3 h and 16 h at 37°C and 5% CO_2_. Cells were lysed with cell lysis buffer (20 mM TrisHCl, pH 8.0, 5 mM EDTA, 40 mM NaCl, and 1% SDS) and kept on ice for 15–30 minutes. The lysate was incubated with proteinase K (20 mg/mL) at 37°C for 2 h. The DNA was precipitated out with absolute ethanol and 5 M NaCl and kept at −20°C overnight. The DNA pellet obtained was washed with 70% ethanol, air dried, and dissolved in sterile distilled water. To visualize the presence of DNA ladder, electrophoresis was performed in 1.8% agarose gel containing ethidium bromide (0.5 *μ*g/mL). DNA marker of 200 bp was run in the same gel. DNA bands were visualized and photographed in a gel documentation system (G: Box, Syngene) [20].

### 2.9. Cell Cycle Analysis by Flow Cytometry

Cell cycle analysis was performed by propidium iodide (PI) based measurements of DNA content of the cell by flow cytometry. DL cells were treated with* D. formosum* ethanolic extract at 250 *μ*g/mL and at the IC_50_ value at 350 *μ*g/mL for 24 h. Cells were harvested after washing with PBS and fixed with 70% alcohol and kept at −20°C. For staining, cells after washing were incubated in 500 *μ*L propidium iodide-(PI-)RNAse solution (1 mg/mL PI solution, Triton X-100 (0.1% v/v), and 10 mg/mL RNAse) for 30 minutes at 37°C using previously described method [21]. Cell cycle was analyzed by FACScan using Cell Quest software (Becton Dickinson).

### 2.10. * In Vivo* Antitumor Activity

The* in vivo *antitumor activity of the extract was evaluated in Dalton's lymphoma bearing mice. DL cells were transplanted by injecting 1 × 10^6^ cells into the peritoneal cavity of control and vehicle groups (6 animals/groups). Similarly, DL cells were transplanted in treated group [III], (A), (B), and (C) with 6 animals/groups. Group I after transplantation was considered as control group without treatment. The day on which transplantation was done was considered as day “0.” After 48 h of transplantation, 100 mg/Kg, 150 mg/Kg, and 175 mg/Kg body weight dose of the ethanolic extracts were administered intraperitoneally on every 3rd day to the DL bearing mice up to the 24th day. DL bearing group II was taken as vehicle group and treated with distilled water every 3rd day up to 24th day.

*Group I.* Control DL bearing mice (without treatment). 

*Group II.* Vehicle group (distilled water). 

*Group III.* Treated group, DL bearing mice.

- With 100 mg/kg body weight of the ethanolic extract treatment.
- With 150 mg/kg body weight of the ethanolic extract treatment.
- With 175 mg/kg body weight of the ethanolic extract treatment.

Evaluation of antitumor activity was performed according to National Cancer Institute (NCI) protocols. This is done by computing treated/control (T/C) value, which is the median survival time of the treated group of animals (T) divided by that of control group (C). The T/C ratio is given as a percentage and a compound/drug is termed as active if it shows T/C value ≥ 120%.

### 2.11. Statistical Analysis

Data are presented as mean ± standard error mean (SEM) of at least three independent experiments and statistical analysis of data was performed with one-way analysis of variance (ANOVA) followed by* Bonferroni t*-test and *P* values < 0.05 were considered significant using Sigma Stat 2.0 version.

## 3. Results and Discussion

### 3.1. MTT Assay

**Figure 1:**
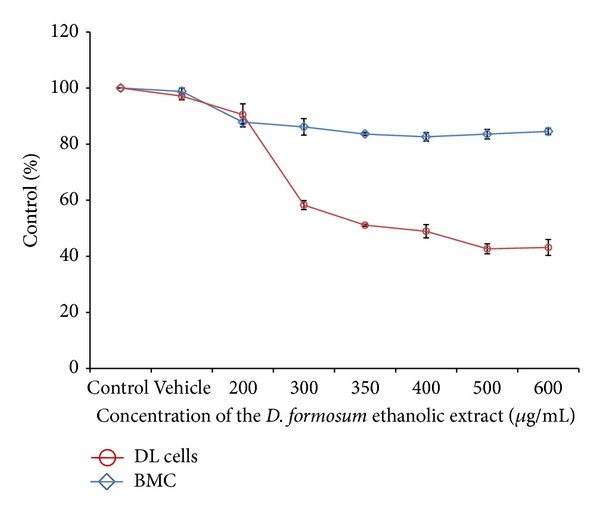
Cell viability was determined by MTT assay. The graph represents the cytotoxicity profile of* D. formosum *ethanolic extract against DL cells and mouse BMC at different concentrations (200 *μ*g/mL–600 *μ*g/mL) on 24 h incubation. Results are expressed as a percentage of control ± SEM from at least three independent experiments.

The ethanolic extract was screened for its cytotoxicity by MTT assay at different concentrations to determine the IC_50_ value in DL cells and in normal mouse bone marrow cells. The ethanolic extract of* D. formosum *induced cytotoxic response in a concentration-dependent manner with an IC_50_ value at 350 *μ*g/mL against DL cells (Figure 1). In the case for normal mouse bone marrow cells the ethanolic extract did not induce cytotoxicity as the IC_50_ value was not obtained even with higher dose of 2 mg/mL. The vehicle group also did not show any effect towards DL cells (Figure 1).

The data obtained from the MTT assay clearly showed that* D. formosum *ethanolic extract can potentially induce cytotoxicity towards DL cells without affecting the normal cells. It was reported that the denbinobin obtained from* Ephemerantha lonchophylla* was found to reduce the cell viability of human colorectal cancer HCT-116 and HT-29 cells in a concentration-dependent manner as measured by MTT assay [22]. In a report in 2012, the IC_50_ with* Curcuma longa* hot water extract was found between 50 and 150 *μ*g/mL on HepG2 cell line [23]. It was also reported that* Piper sarmentosum *ethanolic extract shows a profound effect on a human hepatoma cell line (HepG2) with IC_50_ value at 12.5 *μ*g/mL. In contrast, the ethanolic extract did not induce cytotoxicity in a nonmalignant cell line (Chang's liver cell line) [24]. Therefore we finally illustrate that the ethanolic extract induces cytotoxic effect in DL cells but fails to exert cytotoxicity towards normal cells even at higher concentration.

### 3.2. Cell Morphology Analysis by Fluorescent Staining

**Figure 2:**
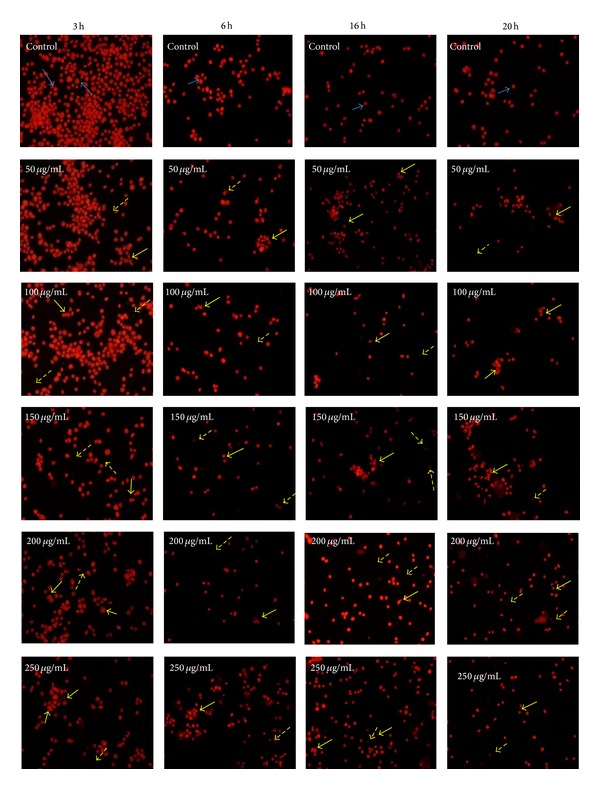
PI staining of nuclei was done to examine morphological changes induced by* D. formosum* ethanolic extract at 50, 100, 150, 200, and 250 *μ*g/mL concentrations and control under fluorescence microscope after treatment for 3 h, 6 h, 16 h, and 20 h, respectively [blue arrow shows live cells, yellow arrow represents apoptotic cells, and dotted yellow arrow shows presence of apoptotic bodies (late stage apoptosis)].

Disruption in apoptotic pathway is considered a major cancer hallmark [25, 26]. Many studies have shown that various anticancer agents exert anticancer property by the process of apoptosis [27, 28]. During apoptotic cell death, there is activation of endogenous nuclease(s) which cleaves DNA into oligonucleosomal fragments. This phenomenon is associated with the appearance of dense and crescent-shaped chromatin aggregates and ultimately leads to the fragmentation of nucleus into dense granular particles (apoptotic bodies) [17]. To identify whether the ethanolic extract induced inhibition of DL cell growth via apoptosis, DNA binding dye, propidium iodide (PI) was used to observe the apoptotic morphology. The PI staining of* D. formosum* ethanolic extract treated DL cells at different concentration and incubation time showed typical apoptotic morphology with brightly red, condensed nuclei (intact or fragmented), and formation of apoptotic bodies compared to the control DL cells with round intact red nucleus (Figure 2). We found that there was a gradual increase of apoptotic cells and apoptotic bodies in dose- and time-dependent manner compared to control. The apoptotic cell morphology was also observed under phase contrast (data not included) but given as supplementary data (see Supplementary Material available online at http://dx.doi.org/10.1155/2014/753451).

Apoptotic induction was also shown in azurin synthesized from* P. aeruginosa* MTCC 2453 in Dalton's lymphoma ascites by PI staining, which showed apoptotic features like condensed nuclei and formation of apoptotic bodies compared to the untreated DL cells [29].

**Figure 3:**
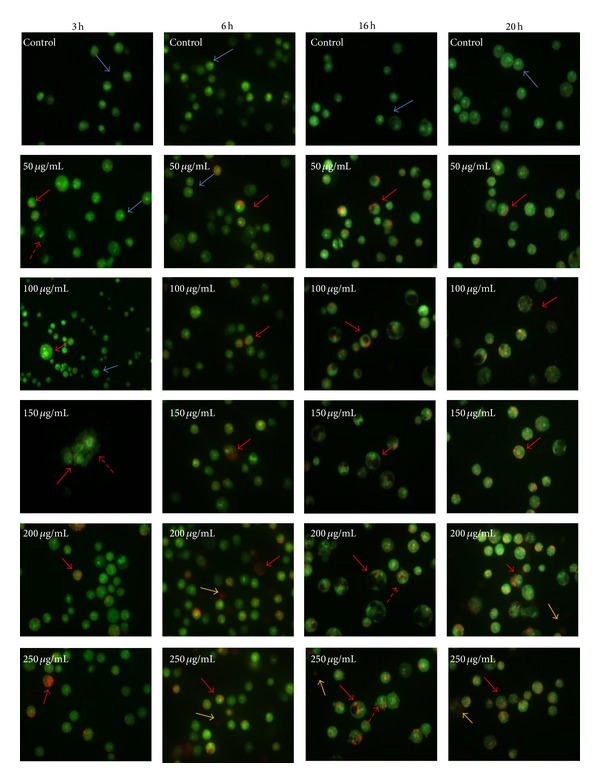
Acridine orange/ethidium bromide staining was carried out to reveal apoptotic cell morphology of DL treated cells with the ethanolic extract at 50, 100, 150, 200, and 250 *μ*g/mL and a control on 3 h, 6 h, 16 h, and 20 h incubation, respectively [blue arrow shows live cells, red arrows show late apoptotic cells, dotted red arrow shows membrane blebbing in early apoptotic cells, and yellow arrows show necrotic cells].

Acridine orange/ethidium bromide (AO/EB) staining was used to observe the apoptotic and necrotic cell nuclear morphology after treatment with the ethanolic extract at different time and concentration (Figure 3). The cells showing green fluorescence with intact green nucleus represent the live cells in control and in treated cells. The morphology of cells was found to be transformed after the treatment, showing cellular shrinkage, membrane blebbing, and typical nuclear fragmentation. We also found that treatment for short duration (3 h) showed early apoptotic cells with more membrane blebbing and yellowish/orange condensed chromatin. But with gradual increase in incubation time (6 h, 16 h, and 20 h) few membrane blebbing and more condensed and fragmented chromatin were observed in most of the cells; at concentrations 200 *μ*g/mL and 250 *μ*g/mL, even presence of necrotic cell was observed with deep orange nucleus which might be due to toxicity with higher dose.

Similarly, morphological examination of HL-60 cell lines after treatment with erianin, a natural product extracted from* Dendrobium chrysotoxum *Lindl., for 24 h showed altered cell morphology-like live cells with normal chromatin and dead cells showed early and late apoptotic cells with condensed and fragmented chromatin [11]. Similar observations in our case indicate that the ethanolic extract of* D. formosum *could possibly induce cell death through an apoptotic pathway.

**Figure 4:**
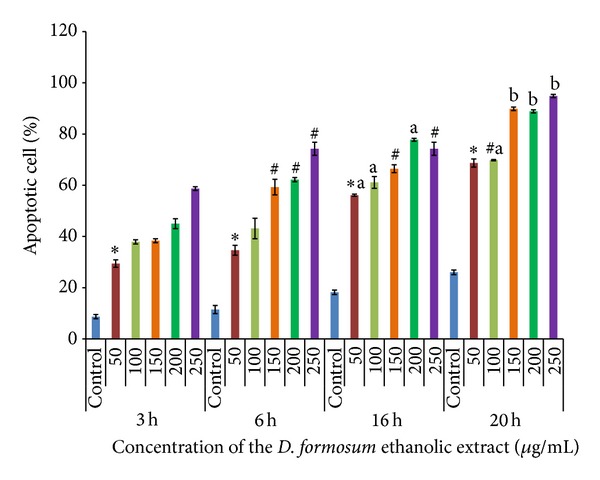
To determine the percentage of apoptotic cells at different times (3 h, 6 h, 16 h, and 20 h) and at different concentrations (50, 100, 150, 200, and 250 *μ*g/mL) acridine orange/ethidium bromide staining was carried out. Data were plotted as percentage of apoptotic cells and represented as mean ± standard error mean (SEM) of at least three independent experiments. “∗” represents significant difference at *P* < 0.05 compared to their respective control. “#” represents significant difference from 6 h, 16 h, and 20 h versus 3 h whereas “a” represents significant difference from 6 h to 16 h and “b” represents significant difference from 16 h to 20 h at *P* < 0.05.

Apoptotic Index Analysis. In the present study, the apoptotic index measured from acridine orange ethidium bromide staining exhibits significant increase in apoptotic cell death after treatment with the ethanolic extract both in dose- and time-dependent manner compared to control which can be interpreted from the histogram (Figure 4). The frequency of apoptotic cells with 3 h of incubation was 29.4% at 50 *μ*g/mL and 58% at 250 *μ*g/mL with respect to 8.6% in control. Similarly with 6 h of incubation the frequency of apoptotic cell was increased to 36% at 50 *μ*g/mL and 72% at 250 *μ*g/mL with 11.8% in control. Further, with 16 h and 20 h samples result in increase of 56% to 68% cell death and 74% to 94% with 50 *μ*g/mL to 250 *μ*g/mL, respectively, in comparison to 19% and 27% with their respective control. Our results illustrate that there is significant increase in apoptotic cell death at 3 h, 6 h, 16 h, and 20 h compared to their respective control and we also observe there is significant increase in apoptotic cells when compared between different times of incubation.

Similar observation by another group showed that the percentage of apoptotic cells in the overall population (apoptotic index) increases significantly in* Piper sarmentosum *ethanolic extract-treated HepG2 cells at 24 h, 48 h, and 72 h compared to controls [24]. Therefore, results from our present study indicate an increase in apoptotic cell population induced by the ethanolic extract in concentration as well as time-dependent manner.

### 3.3. DNA Fragmentation Assay

**Figure 5:**
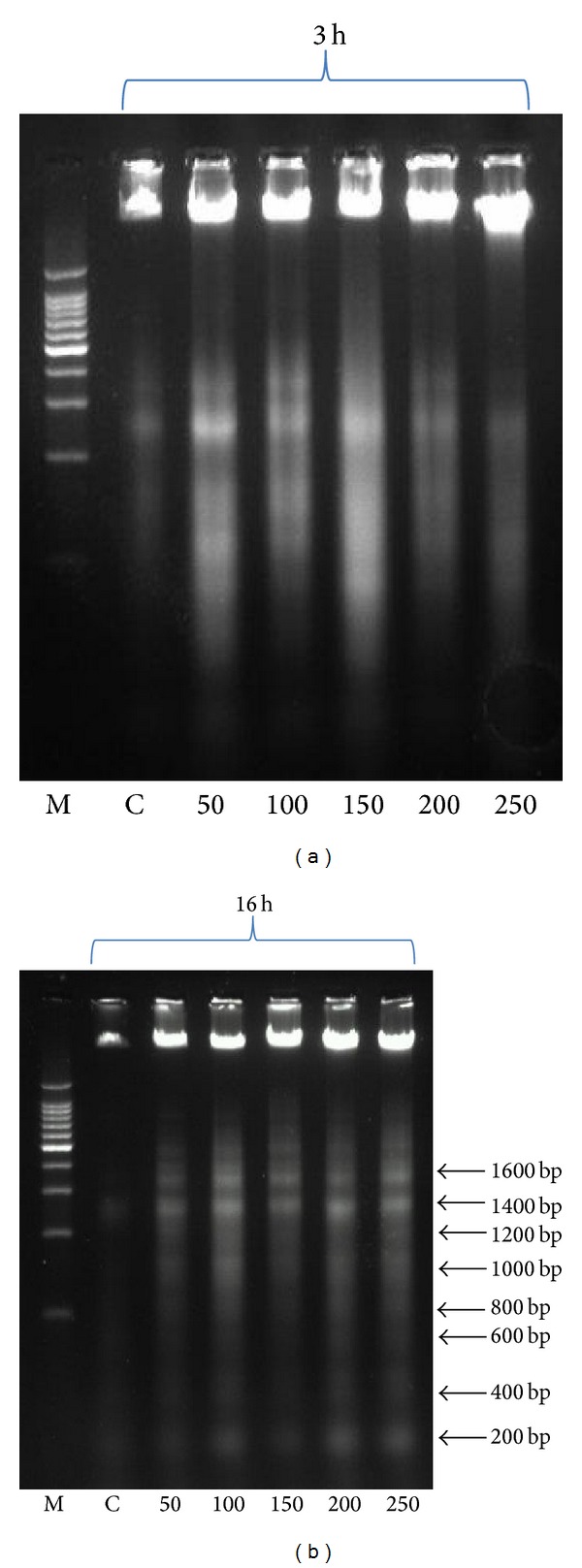
1.8% agarose gel electrophoresis was run to separate DNA fragments after incubation of DL cells with various concentrations (50, 100, 150, 200, and 250 *μ*g/mL) of ethanolic extract for (a) 3 h and (b) 16 h, respectively. DNA fragments separated were visualised under UV light. M: 200 bp DNA ladder marker, C: control. Three independent experiments were performed (*n* = 3).

To explicate the apoptotic induction by the ethanolic extract in cancer cells, DNA fragmentation assay was done in DL cells. DNA fragmentation is one of the archetypal biochemical features of apoptosis. During late stage of apoptosis, nuclear DNA is cleaved at an interval of 180–200 base pairs (bp) by endonucleases and due to this DNA bands appear like ladder on an agarose gel. DNA fragmentation was analysed after treatment with the ethanolic extract at 3 h and 16 h. In case of 3 h incubation less laddering pattern was obtained which might be due to the presence of early apoptotic cells (membrane blebbing) with less nuclear fragmentation as shown in Figure 5(a) when compared to control, whereas it produced a typical ladder-like pattern at 16 h incubation, shown in Figure 5(b), confirming characteristic nuclear fragmentation in the late stage of apoptosis, which can also be inferred from the result of AO/EB fluorescence staining. Therefore, we can conclude that, with short incubation time, the ethanolic extract brings about early apoptosis, but when incubated for longer duration, later stage of apoptotic cells with more nuclear fragmentation was observed.

Our data are also in corroboration with the findings of Mishra et al. who reported a DNA ladder pattern with aqueous ethanol seed extract of* Ziziphus mauritiana *in HL-60 cells in a concentration-dependent manner and a time-dependent study showed typical ladder pattern due to induction of apoptosis [30]. Yang et al. in 2005 reported the presence of DNA ladder with denbinobin using DNA fragmentation assay at higher concentration in COLO 205 cells but at lower concentrations DNA ladder was not observed [31]. Erianin also showed same pattern of DNA fragmentation on 24 h treatment in HL-60 cell lines [11]. The result obtained from our experiment finally confirms the cell death via apoptosis by the* D. formosum* ethanolic extract. Thus, this illustrates that the anticancer effect of the ethanolic extract may act through the apoptotic signalling.

### 3.4. Cell Cycle Analysis

**Figure 6:**
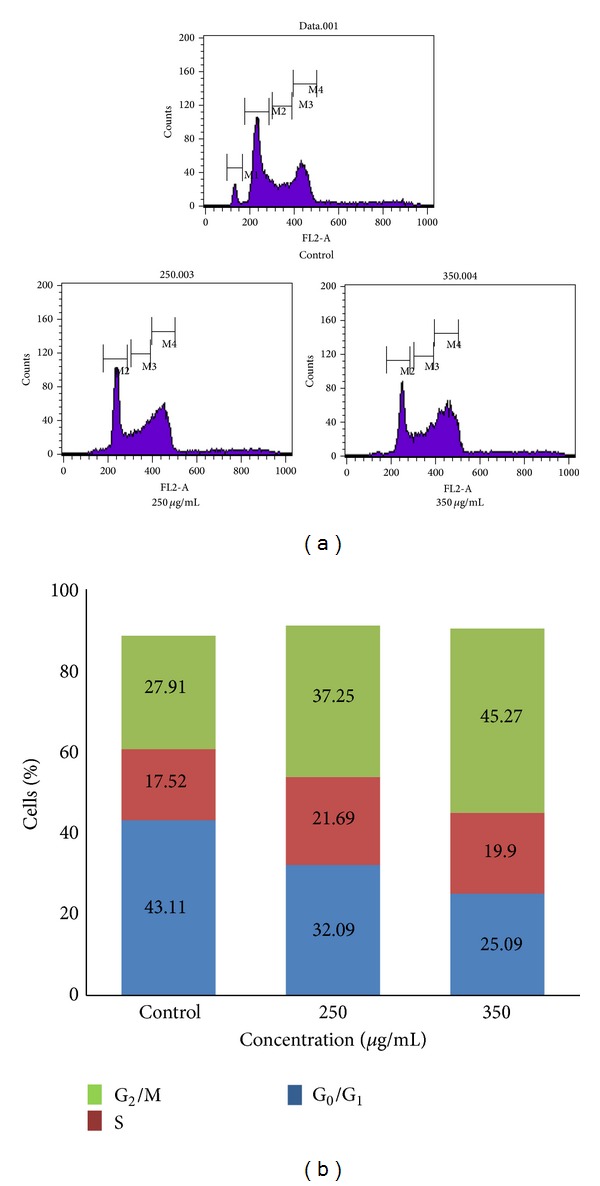
(a) Cell cycle analysis in DL cells after* D. formosum* ethanolic extract treatment was done by flow cytometry. DL cells were incubated with the ethanolic extract (250 and 350 *μ*g/mL) for 24 h and propidium iodide (PI) staining was done to determine the DNA content. (b) The graph shows the percentage of DL cells in different phases of cell cycle on incubation with* D. formosum* ethanolic extract at 250 and 350 *μ*g/mL for 24 h as analyzed by flow cytometry.

To clarify whether* D. formosum* exert antitumor effect only by inducing apoptosis or they also induce cell cycle arrest, we assessed cell cycle distribution by FACS analysis in asynchronous DL cells. The effect of ethanolic extract on DL cell cycle is represented in Figures 6(a) and 6(b). Our results showed that the ethanolic extract induced arrest of the cell cycle at the G_2_/M phase. Treatment with the ethanolic extract at 250 *μ*g/mL and 350 *μ*g/mL shifted the population of DL cells into the G_2_/M phase from G_1_ phase. At 250 *μ*g/mL, 37% cells were in G_2_/M phase while, at 350 *μ*g/mL, cells in the G_2_/M phase were increased to 45% with reduced number of cells at G_1_ phase as compared to control (Figures 6(a) and 6(b)). The G_2_/M cell cycle checkpoint blocks the entry of cells into mitotic stage when DNA is damaged [32]. p53 can regulate the G_2_/M transition through the induction of apoptosis [33, 34]. Moscatilin from Indian orchid* Dendrobium moschatum* and* Dendrobium loddigesii* induces a time-dependent arrest of asynchronized HCT-116 cells at G_2_/M phase as reduced numbers of cells were present at the G_1_ phase followed by increased number of cells at the sub-G_1_ phase [35]. Armania et al. in 2013 suggested that the active fractions of* Dillenia suffruticosa* extract exert anticancer activity by inducing apoptosis and cell cycle arrest at G_2_/M phase in MCF-7 cells [36].

Thus, from our observation we could say that the ethanolic extract induces arrest of cell cycle at G_2_/ M phase, but role of different proteins involved in the induction of G_2_/M arrest in DL cells still needs further investigation.

### 3.5. *In Vivo* Antitumor Activity

**Figure 7:**
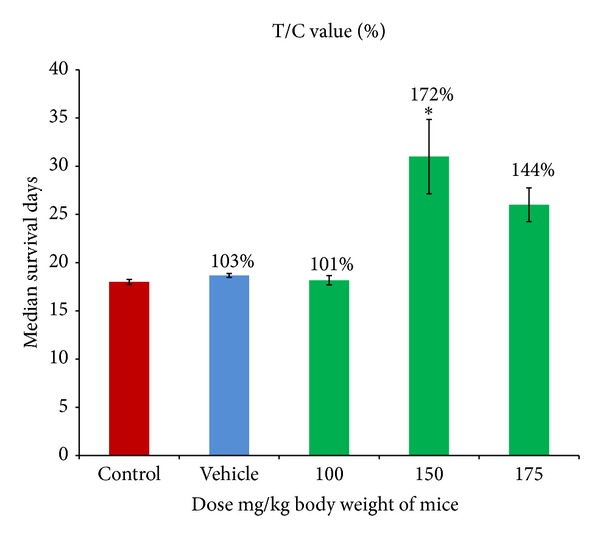
Evaluation of antitumor activity of theethanolic extract against Dalton's lymphoma transplanted mice on 100, 150, and 175 mg/Kg body weight treatment was done by computing median survival time. Data were plotted for median survival days and represented as mean ± standard error mean (SEM) of at least three independent experiments. ∗ represents significant difference at (*P* < 0.05) compared to control. The median survival time of the treated group of animals (T) divided by control group (C) is given as a percentage and an effective T/C value is considered when the T/C ratio is ≥120% (according to NCI).

The* in vivo* antitumor activity of the ethanolic extract was measured after the transplantation of Dalton's lymphoma in Swiss albino mice. The result of the* in vivo* antitumor activity was expressed as ratio of the median survival days of the treated and control group (T/C) of DL bearing mice on treatment with the ethanolic extract. The data obtained has been shown as histogram (Figure 7). An effective T/C value of 172% with significant increase in life span compared to control was obtained when the mice were treated with 150 mg/Kg body weight of the ethanolic extract thereby increasing the survivability of tumour bearing mice, whereas the T/C value reduced to 144% on treating the mice with 175 mg/Kg. This result indicates that higher dose of treatment with the ethanolic extract may induce toxicity, resulting in the decrease in survivability of tumour bearing mice. The T/C value (101%) was found to be ineffective when treatment was done with less than 150 mg/Kg body weight (100 mg/Kg). Treatment with water in vehicle group did not increase the survival time of DL bearing mice with T/C value of only 103%. The result obtained shows that treatment with 150 mg/Kg body weight is most effective in increasing the survivability of the mice thereby delaying the tumour growth.

A moderate growth delay in two types of tumors Bel7402 and melanoma A375 after treatment with 100 mg/Kg body weight of erianin, a natural product from an orchid* Ephemerantha lonchophylla, *was shown [37]. Similarly, treatment with 50 mg/Kg denbinobin significantly reduced tumour growth up to 68% in nude mice bearing COLO 205 tumor xenograft [31]. The effective T/C value obtained from our experiment shows that the ethanolic extract elicits good antitumor activity by increasing median survival time of the DL bearing mice.

## 4. Conclusions

The results obtained involving* in vitro *and* in vivo *studies in Dalton's lymphoma cells clearly demonstrated potent anticancer activity of ethanolic extract of* Dendrobium formosum*. This anticancer activity is due to apoptotic inducing property and cell cycle delay with the plant extract thereby enhancing the survivability of the tumour bearing mice. The fundamental advantage of this ethanolic extract is that it exhibits high cytotoxicity towards Dalton's lymphoma cells without affecting normal cells. Thus, further investigation, including the isolation of the bioactive components in* Dendrobium formosum*, may be necessary to improve the efficacy of the extract. The results from our present study are found to be promising, which should be followed by the identification of the molecular mechanism regulated by the ethanolic extract to combat cancer.

## Supplementary Material

Cell Morphology Analysis by Light microscopy.
DL cells were isolated from DL bearing mice. 1x10^6^cells/mL (DL) were treated with 50*μ*g/mL, 100*μ*g/mL, 150*μ*g/mL, 200*μ*g/mL and 250*μ*g/mL of the D. formosum ethanolic extract. The treated cells were incubated for 3h, 6h, 16h and 20h at 37°C and 5% CO2. After the incubation cells were washed in PBS. After washing, the cells were re-suspended in PBS. The cells were then examined on a slide in phase contrast under a light microscope and the images were captured.
We observed altered cell morphology in the treated DL cells compared to control cells. The images indicate a dose and time dependent increase in the presence of apoptotic cells compared to control DL cells. Images attached below.
