# Supplementary material for: Antitumor Activity of Ethanolic Extract of Dendrobium formosum in T-Cell Lymphoma: An In Vitro and In Vivo Study
Source: Biomed Res Int. 2014 May 18;2014:753451. doi: 10.1155/2014/753451 (PMC4052125; doi:10.1155/2014/753451)
Supplement: Supplementary file 1 — Cell Morphology Analysis by Light microscopy. DL cells were isolated from DL bearing mice. 1x10^6^cells/mL (DL) were treated with 50μg/mL, 100μg/mL, 150μg/mL, 200μg/mL and 250μg/mL of the D. formosum ethanolic extract. The treated cells were incubated for 3h, 6h, 16h and 20h at 37°C and 5% CO2. After the incubation cells were washed in PBS. After washing, the cells were re-suspended in PBS. The cells were then examined on a slide in phase contrast under a light microscope and the images were captured. We observed altered cell morphology in the treated DL cells compared to control cells. The images indicate a dose and time dependent increase in the presence of apoptotic cells compared to control DL cells. Images attached below. [file 753451.f1.pdf]

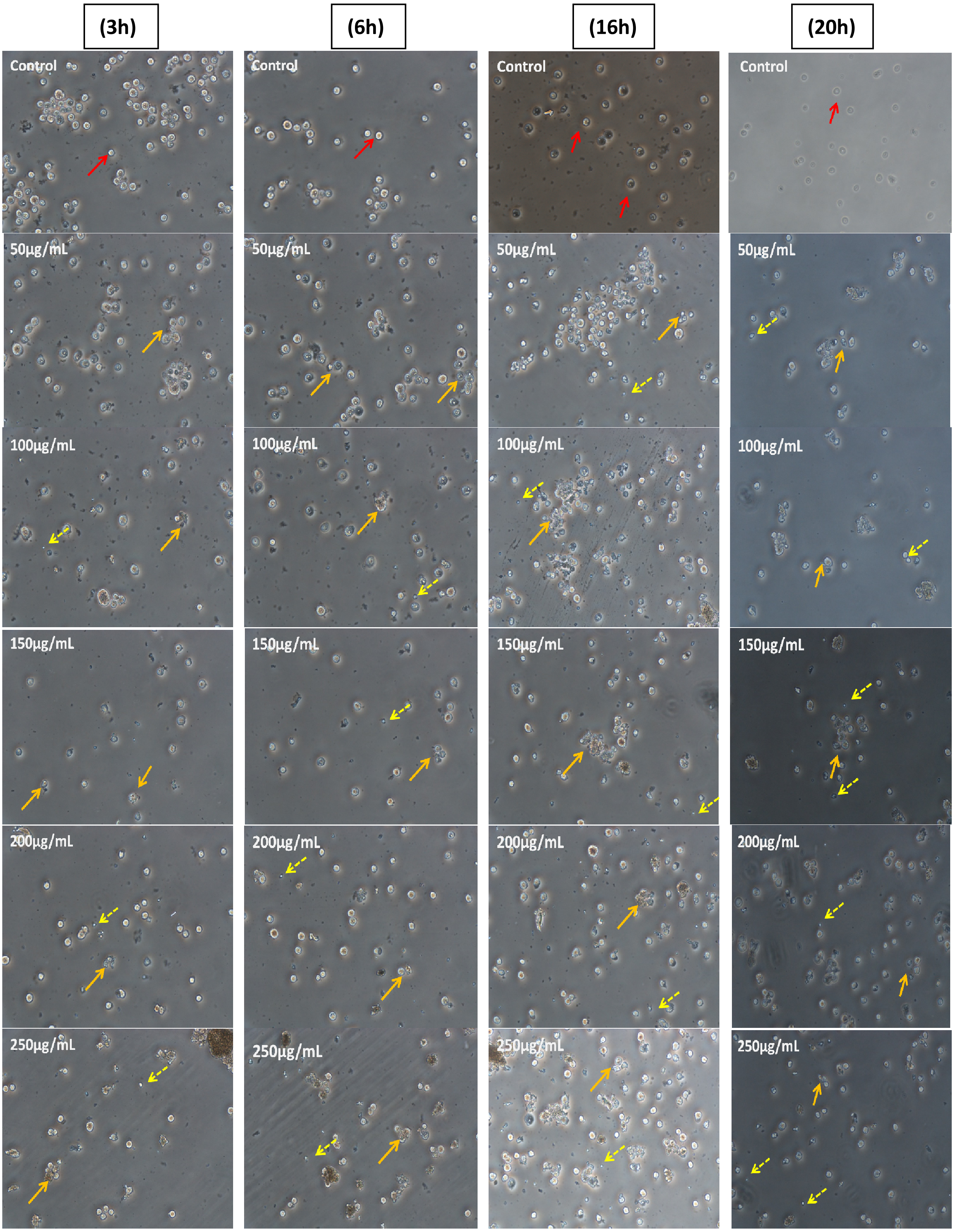

Fig.1. Phase contrast microscopy images of DL cells showing morphological changes induced by *D. formosum* ethanolic extract at 50, 100, 150, 200 and 250µg/mL concentration and control for 24h. Red arrow shows live cells, yellow arrow represent apoptotic cells and dotted yellow arrow shows presence of apoptotic bodies (late stage apoptosis)].
